# A genomic approach to coral-dinoflagellate symbiosis: studies of *Acropora digitifera* and *Symbiodinium minutum*

**DOI:** 10.3389/fmicb.2014.00336

**Published:** 2014-07-07

**Authors:** Chuya Shinzato, Sutada Mungpakdee, Nori Satoh, Eiichi Shoguchi

**Affiliations:** Marine Genomics Unit, Okinawa Institute of Science and Technology Graduate UniversityOkinawa, Japan

**Keywords:** corals, symbiosis, *Symbiodinium*, genome, transcriptome

## Abstract

Far more intimate knowledge of scleractinian coral biology is essential in order to understand how diverse coral-symbiont endosymbioses have been established. In particular, molecular and cellular mechanisms enabling the establishment and maintenance of obligate endosymbiosis with photosynthetic dinoflagellates require further clarification. By extension, such understanding may also shed light upon environmental conditions that promote the collapse of this mutualism. Genomic data undergird studies of all symbiotic processes. Here we review recent genomic data derived from the scleractinian coral, *Acropora digitifera*, and the endosymbiotic dinoflagellate, *Symbiodinium minutum*. We discuss *Acropora* genes involved in calcification, embryonic development, innate immunity, apoptosis, autophagy, UV resistance, fluorescence, photoreceptors, circadian clocks, etc. We also detail gene loss in amino acid metabolism that may explain at least part of the *Acropora* stress-response. Characteristic features of the *Symbiodinium* genome are also reviewed, focusing on the expansion of certain gene families, the molecular basis for permanently condensed chromatin, unique spliceosomal splicing, and unusual gene arrangement. Salient features of the *Symbiodinium* plastid and mitochondrial genomes are also illuminated. Although many questions regarding these interdependent genomes remain, we summarize information necessary for future studies of coral-dinoflagellate endosymbiosis.

## Introduction

Coral reefs and tropical forests are places that foster the greatest diversities of organisms on the earth. Even though coral reefs occupy only ~1% of the seas, they are estimated to harbor around one-third of all described marine species (Wilkinson, 2004), and their productivity supports around one quarter of marine fisheries. However, due to human activities and climate changes, reefs decline in abundance, and wholesale loss of reef habitats is one of the most pressing environmental issues of our time.

The major architects of coral reefs, the scleractinian corals, are anthozoan cnidarians that form obligate endosymbioses with photosynthetic dinoflagellates of the genus *Symbiodinium*. The symbionts confer upon the coral holobiont the ability to fix CO_2_ and to deposit the massive aragonite (a form of calcium carbonate) skeletons that distinguish reef-building corals from other anthozoans, such as sea anemones. The association is fragile however, collapsing under stress and from disease. Molecular and cellular mechanisms underlying much of coral biology, including the establishment, maintenance, and breakdown of coral-*Symbiodinium* symbioses remain to be elucidated.

In order to investigate mechanisms that support this mutualism, genomic information from both corals and *Symbiodinium* is essential. Proteomics approaches have also been applied to coral and *Symbiodinium* studies (Drake et al., 2013; Ramos-Silva et al., 2013). Following cloning and characterization of single genes (e.g., Berghammer et al., 1996; Miller et al., 2000), the first large molecular dataset available for a coral was a collection of ~3000 expression sequence tags (ESTs) from the Indo-Pacific complex coral, *Acropora millepora* (Kortschak et al., 2003). Since then, several EST data sets and transcriptomics studies in corals, as well as *Symbiodinium* spp. have appeared (Tables 1, 2). In 2011, a draft genome of *Acropora digitifera* was decoded (Table 1) (Shinzato et al., 2011). Then, in 2013, a draft genome of *Symbiodinium minutum* was decoded (Table 2) (Shoguchi et al., 2013a). The present review describes characteristic features of these two genomes, with the hope that this information may support future studies of coral biology.

**Table 1:** **Published genomics and transcriptomics datasets of scleractinian corals**.

**Dataset**	**Species**	**Sequencing technologies**	**References**
Genome	*Acropora digitifera*	454, Illumina	Shinzato et al., 2011
Transcriptome	*Acropora millepora*	Sanger, 454, Illumina	Moya et al., 2012
	*Acropora hyacinthus*	Illumina	Barshis et al., 2013
	*Acropora palmata*	Sanger, 454	Polato et al., 2011
	*Acropora cervicornis*	Illumina	Libro et al., 2013
	*Porites australiensis*	Illumina	Shinzato et al., 2014
	*Porites astreoides*	454	Kenkel et al., 2013
	*Favia* sp.	Illumina	Mehr et al., 2013
	*Montastraea faveolata*	Sanger	Schwarz et al., 2008
	*Stylophora pistillata*	454	Karako-Lampert et al., 2014
	*Pocillopora damicornis*	454	Traylor-Knowles et al., 2011

**Table 2:** **Published genomics and transcriptomics datasets of *Symbiodinium***.

**Dataset**	**Species (strain ID)**	**Clade**	**Host**	**Sequencing technologies**	**References**
Genome	*Symbiodinium minutum* (Mf 1.05b.01)	B1	*Montastraea faveolata*	454, Illumina	Shoguchi et al., 2013a
Transcriptomes	*Symbiodinium minutum* (Mf 1.05b.01)	B1	*Montastraea faveolata*	Illumina	Shoguchi et al., 2013a
	*Symbiodinium microadriaticum* (CCMP2467)	A1	*Stylophora pistillata*	Illumina	Baumgarten et al., 2013
	*Symbiodinium* spp.	C	*Acropora hyacinthus*	Illumina	Ladner et al., 2012
	*Symbiodinium* spp.	D	*Acropora hyacinthus*	Illumina	Ladner et al., 2012
	*Symbiodinium* sp. (Mf1.05b)	B1	*Montastraea faveolata*	454	Bayer et al., 2012
	*Symbiodinium* sp. (CassKB8)	A	*Cassiopea* sp.	454	Bayer et al., 2012
	*Symbiodinium* sp.	C3K	*Acropora hyacinthus*	Illumina	Barshis et al., 2014^*^
	*Symbiodinium* sp.	D2	*Acropora hyacinthus*	Illumina	Barshis et al., 2014^*^
	*Symbiodinium* sp.	C15	*Porites australiensis*	Illumina	Shinzato et al., 2014^*^
	*Symbiodinium kawagutii* (CCMP2468)	F1	*Montipora verrucosa*	Sanger	Zhang et al., 2013
	*Symbiodinium* sp.	A	*Aiptasia pallida*	Sanger	Sunagawa et al., 2009
	*Symbiodinium* sp. (CassKB8)	A	*Cassiopea* sp.	Sanger	Voolstra et al., 2009
	*Symbiodinium* sp.	C3	*Acropora aspera*	Sanger	Leggat et al., 2007

* *From a mixed host/symbiont cDNA library*.

## The *acropora digitifera* genome

**Figure 1:**
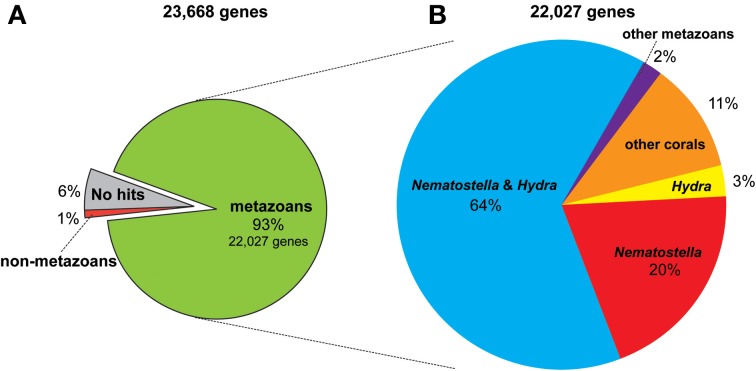
**The proportion of similarity of *Acropora digitifera* gene models to those of other metazoans (see text for the method). (A)** The 93% (22,027) of the 23,668 gene models have similarity to other metazoans, while 1% of them for non-metazoans and 6% show no similarity to proteins deposited in NCBI database (NR). **(B)** Of the 22,027 metazoan-similarity genes, 64% had counterparts in both *Nematostella* and *Hydra* genomes, 20% of them for *Nematostella* but not in *Hydra*, 3% for *Hydra* but not in *Nematostella*, and 2% for other metazoans but not in *Nematostella* and *Hydra*. Eleven percent of them have corresponding ESTs of corals reported in NCBI.

The genome of *A. digitifera*, decoded using next-generation sequencing technology, is ~420-Mbp in size, 39% G+C, and contains 23,668 predicted protein-coding loci (Shinzato et al., 2011). The coral gene set is comparable in size and composition to those of *Nematostella vectensis* (Putnam et al., 2007) and *Hydra magnipapillata* (Chapman et al., 2010). The *A. digitifera* genome browser is accessible at http://marinegenomics.oist.jp/acropora_digitifera (Koyanagi et al., 2013). Approximately 93% of *A. digitifera* genes have homologs in other metazoans (Figure 1A), and of these, 11% have significant homology only amongst EST data from corals (Figure 1B) (Hemmrich and Bosch, 2008), suggesting the presence of a considerable number of coral-specific genes. As discussed later, the *Acropora* nuclear DNA sequences do not contain any *Symbiodinium*-related genome sequences.

### Evolutionary origins of reef-building corals

Corals are morphologically very similar to sea anemones, but their evolutionary origins are obscure. Reef building scleractinians first appeared in the fossil record in the mid Triassic (~240 MYR) (Stanley and Fautin, 2001), but were already highly diversified, suggesting much earlier origins. The availability of fully sequenced genomes for three cnidarians (*Acropora, Nematostella*, and *Hydra*) allows us to estimate the time of divergence between corals and other metazoans. Molecular phylogenetic analyses, based on an alignment of 94,200 amino acids, suggest a divergence time of 520 ~ 490 MYR for *Acropora* and *Nematostella* (late Cambrian or early Ordovician). This implies early origin of Scleractinia indicates that corals have persisted through previous periods of dramatic environmental change, including the mass extinction event at the Permian/Triassic boundary, when global CO_2_ and temperature were much higher than at present. However, molecular phylogeny of symbiotic dinoflagellates suggests that *Symbiodinium* originated in early Eocene, and that the majority of extant lineages diversified since Mid-Eocene, ~18 MYR ago (Pochon et al., 2006). Therefore, it is far from certain that modern coral reefs can adapt to the rapid environmental changes now occurring.

### Traces of symbiosis in the coral genome

Obligate endosymbiosis of corals dates from at least the mid Triassic (Stanley and Fautin, 2001), and the longevity of this association might be expected to have resulted in changes in the coral genome. However, a comprehensive search of *Acropora* nuclear DNA sequences failed to find any *Symbiodinium* DNA sequences (Shinzato et al., 2011); hence there is, as yet, no evidence for horizontal gene transfer from symbiont to host. Neither is *Symbiodinium* vertically transferred via host gametes. As a result, the symbiosis must be re-established with each generation. Nonetheless, comparative analyses imply that *Acropora* is probably metabolically dependent upon its endosymbiont.

When the metabolic repertoire of *A. digitifera* was compared using the KEGG pathway database to that of its non-symbiotic relative, *Nematostella*, it became apparent that *Acropora* lost a gene for cysteine biosynthesis. Biosynthesis of cysteine from homocysteine and/or serine requires two enzymes, cystathionine beta-synthase (Cbs) and cystathionase (cystathionine gamma-lyase) (Table 3). Although both the *A. digitifera* and *Nematostella* genomes encode cystathionase, the gene for Cbs could not be identified in *Acropora* despite the existence of an ortholog in *Nematostella* (Table 3). An extensive search of transcriptomic data available for various *Acropora* spp. (Hemmrich and Bosch, 2008) failed to identify a *Cbs* transcript in any congener. Moreover, whereas a PCR strategy confirmed the presence of *Cbs* in some other corals (*Galaxea fascicularis, Favites chinenis, Favia lizardensis*, and *Ctenactis echinata*), no amplification products could be obtained for two different *Acropora* species (Table 3). Although further studies of biosynthetic pathways are required, this finding raises the intriguing possibility of a metabolic basis for the obligate nature of symbiosis in *Acropora*. Differences in dependency could potentially explain not only the phenomenon of symbiont selectivity, but also the high sensitivity of *Acropora* to environmental challenges.

**Table 3:** **The presence or absence of a gene encoding cystathionine β-synthase (Cbs) for L-cysteine biosynthesis in corals**.

	**L-Homo cysteine + L-Serine**	***Cbs* →**	**L-Cysta thionine**	***Cth* →**	**L-Cysteine**
*Hydra magnipapillata*		Yes^a^		Yes	
*Nematostella vectensis*		Yes^a^		Yes	
**COMPLEXA**					
*Acropora digitifera*		–^b^		Yes	
*Acropora tenuis*		–^c^		ND	
*Acropora millepora*		–^d^		Yes	
*Galaxea fascicularis*		Yes^c^		ND	
**ROBUSTA**					
*Montastraea faveolata*		Yes^d^		Yes	
*Favia lizardensis*		Yes^c^		ND	
*Favites chinensis*		Yes^c^		ND	
*Ctenactis echinata*		Yes^c^		ND	

*ND, not determined*.

a *Supported by sequenced genome and EST analyses*.

b *Supported by sequenced genome, EST, and PCR amplification of genome DNA*.

c *Supported by PCR-amplification of genome DNA*.

d *Supported by EST analyses*.

### Genes involved in calcification

The coral gene repertoire, with predicted roles in skeleton deposition, is of particular interest, given the likely impact of ocean acidification resulting from rising atmospheric CO_2_ on coral calcification. Surveys of the *Acropora* genome reveal the presence of genes for specific groups of proteins associated with calcification, including the eukaryotic carbonic anhydrases (Jackson et al., 2007). In general, the soluble fraction of the organic matrix (OM) in invertebrates is very rich in acidic amino acids, and has a particularly high aspartic acid composition (Sarashina and Endo, 2006). A number of candidate OM protein genes are present in the *Acropora* genome. Galaxins, first purified from the coral, *G. fascicularis*, are unique to corals and are the only coral skeletal matrix protein for which the complete primary structure has been determined (Fukuda et al., 2003). However, galaxin possesses neither acidic regions (the fraction of Asp+Asn in the galaxin is only 9.7%) nor obvious Ca^2+^ binding domains. Four genes encoding galaxin-related proteins have been identified in the *A. digitifera* genome, including two likely *A. digitifera* homologs of galaxin.

### Transcription factor genes and signaling molecule genes

Cnidarians have genes for transcription factors and signaling molecules comparable to those found in bilaterians (Technau et al., 2005; Putnam et al., 2007) and this is also true of corals (Shinzato et al., 2011). Of those, genes for Hox cluster and basic helix-loop-helix (bHLH) families have been examined in detail in the *A. digitifera* genome.

#### Hox genes

**Figure 2:**
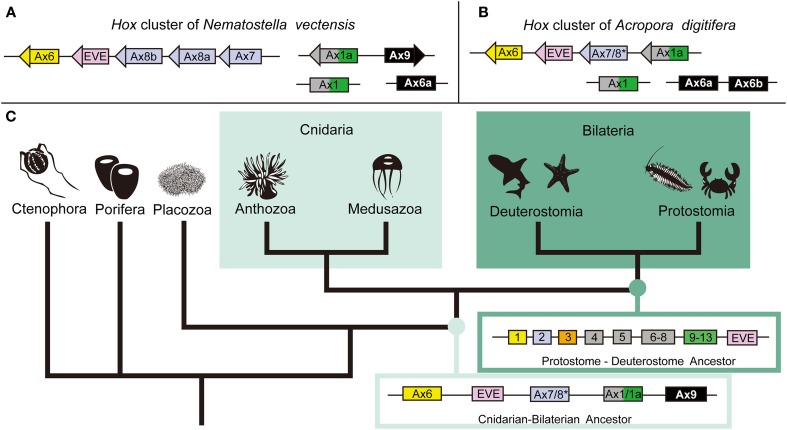
**The anthozoan complement of Hox genes and the implications of the evolution of the Hox cluster**. Comparing the genomic linkage of Hox genes in the sea anemone *N. vectensis* and the staghorn coral *A. digitifera* confirms that cnidarians once had a Hox cluster that contained both anterior and posterior/central class Hox genes. **(A)** The Hox cluster of *N. vectensis* includes the anterior Hox genes Anthox6 (PG1), Anthox8b (PG2), Anthox8a (PG2), and Anthox7 (PG2) as well as the Eve homeobox gene. **(B)** The Hox cluster of *A. digitifera* includes the anterior Hox genes Anthox6 (PG1) and Anthox7/8 (PG2), and the posterior/central class Hox gene Anthox1a (PG4–14), as well as the Eve homeobox gene. Another gene HlxB9 (also named MNX) is found upstream of Anthox6 in the Hox cluster of both genomes (data not shown). **(C)** The metazoan tree of life with inferred ancestral Hox clusters. The ancestor to protostomes and deuterostomes is thought to have had two anterior class Hox genes (Hox1 and Hox2), one paralagous group 3 gene (Hox3), three central class genes (Hox4, Hox5, and Hox6–8), one posterior class Hox gene (Hox9–14), and one Eve homeobox gene. Because of the extended cluster in A. digitifera, we can now say that the cnidarian–bilaterian ancestor had, at least, two anterior class Hox genes (Anthox6 and Anthox7/8), a central/posterior class Hox gene (Anthox1/1a), and the Eve homeobox gene. It is unclear at what point the genomic rearrangement involving the Eve homeobox gene occurred. The origin of the PG3 Hox genes also is not clear. ^*^Anthox7/8 has been categorized as a PG2 Hox gene in previous publications, but it is possible, based on our current phylogenetic analysis, that Anthox7/8 descended from a Hox gene that was lost in bilaterians. Based on the genomic orientation of these genes, we also believe the ancestor likely had a fourth Hox gene potentially related to Anthox9. For more detail information, please see DuBuc et al. (2012). Abbreviations: PG, paralogous group; Ax, Anthox.

*Hox* genes are homeobox transcription factors that play a critical role in developmental patterning (McGinnis et al., 1984). They have been identified in every extant phylum except the Porifera, Ctenophora, and Placozoa. Cnidarians are the only non-bilaterian phylum with *Hox* genes; therefore they are critical to our understanding of early *Hox* cluster evolution. However, the *H. magnipapillata* genome shows no *Hox* cluster (Chapman et al., 2010) and clustering in *N. vectensis* is limited to anterior Hox genes (Chourrout et al., 2006; Putnam et al., 2007; Ryan et al., 2007), raising the question of the degree of *Hox* gene clustering in cnidarians. The *A. digitifera* genome has the most extensive *Hox* cluster reported in any cnidarian (DuBuc et al., 2012). Phylogenetic analysis revealed a total of six *Hox*, one *ParaHox*, three *Mox*, one *Eve*, and one *HlxB9* gene in the *Acropora* genome. Of the six *Hox* genes, two anterior (PG1 and PG2) linked to an *Eve* homeobox gene and an *Anthox1A* gene (Figure 2). Therefore, the *Hox* cluster of the cnidarian–bilaterian ancestor was more extensive than previously thought. These facts are congruent with the existence of an ancient set of constraints on the *Hox* cluster and reinforce the importance of incorporating a wide range of animal species to reconstruct critical ancestral nodes.

#### bHLH genes

bHLH proteins constitute a large group of transcription factors that comprise a basic region for DNA binding and two α-helices, interrupted by a variable loop region, for dimerization. bHLH proteins homo- or heterodimerize to recognize and bind specific core hexa-nucleotides, and play pivotal roles in cell differentiation and proliferation (Massari and Murre, 2000; Jones, 2004). A putative full set of bHLH genes has been described in the genomes of a number of metazoans, and molecular phylogenetic analyses have identified 45 orthologous families of bHLH factors, which were categorized into six high order groups (Atchley and Fitch, 1997).

The *A. digitifera* genome contains a nearly full set of 70 bHLH transcription factors, comparable to the 68 bHLH genes in *N. vectensis* (Gyoja et al., 2012). The *Acropora* genes have been assigned to 29 previously reported orthologous families. In addition, three novel HLH orthologous families have been identified, designated pearl, amber, and peridot (Gyoja et al., 2012). Pearl and amber orthologs are present in genomes and ESTs of the Mollusca and Annelida, in addition to the Cnidaria. Peridot orthologs are present in genomes and ESTs of the Cephalochordata and the Hemichordata, in addition to the Cnidaria. These three genes have apparently been lost in the clades of *Drosophila, Caenorhabditis*, and *Homo sapiens*. Therefore, cnidarians provide information about alteration of transcription factor genes during animal evolution.

### Innate immunity

**Figure 3:**
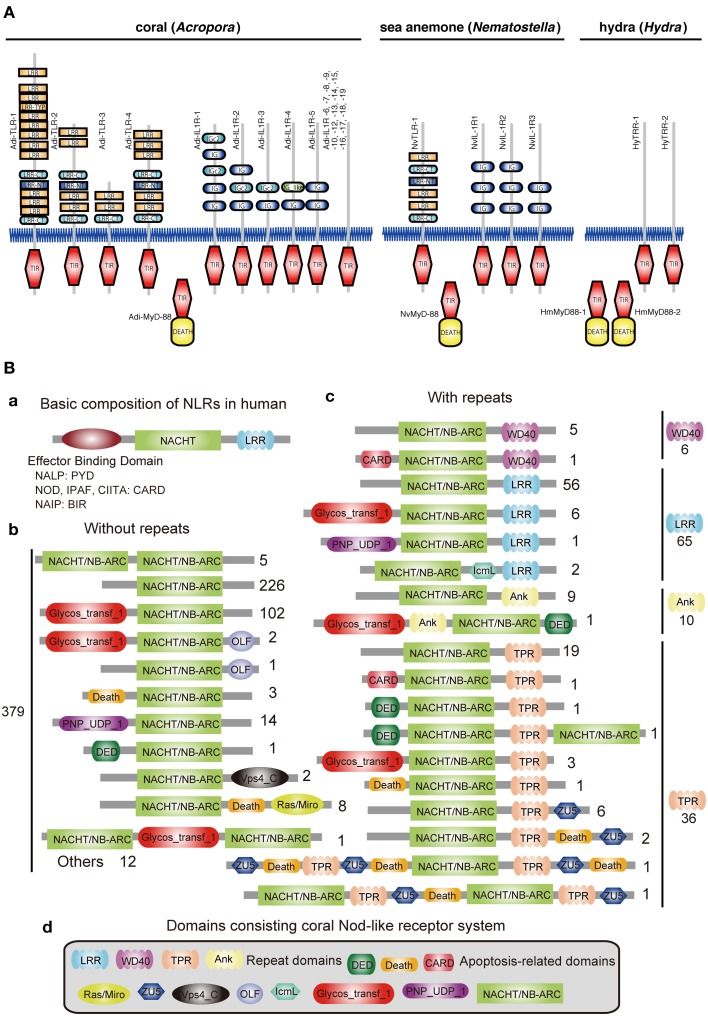
**Innate immunity of corals. (A)** Repertoires of TIR domain-containing proteins of three cnidarians. The schematic representation of the domain structures of all of the TIR domain-containing proteins identified in *Acropora digitifera*, alongside the corresponding complements from *Nematostella vectensis* and *Hydra magnipapillata*. The repertoire of Toll/TLR, IL-1R-like, and TIR-only proteins is significantly more complex in the case of *A. digitifera* than in *N. vectensis* or *H. magnipapillata*. TIR, TIR domain; DEATH, DEATH domain; IG and IGc2, Ig domain; LRR, LRY-TRY, LRR-CT and LRR-NT, leucine-rich repeats. **(B)** The complexity of the NBD repertoire of *Acropora digitifera*. The figure summarizes the numbers of loci with each kind of domain architecture. (a) The basic composition of NRLs in human is, from N- to C-terminus, effector binding domain, NACHT domain and repeats (LRR). The effector binding domain components are PYD in NALPs, CARD in NODs, IPAF, CIITA and BIR in NAIP. (b) A total of 379 coral NBD loci do not encode repeat domains. Numbers to the right of schematics represent the number of loci with each specific architecture. (c) In addition, 117 loci in the coral encode NBDs and repeat domains of the WD40, LRR, Ank, or TPR types. (d) The various domains identified in the Nod-like proteins of *Acropora*.

Innate immunity in corals is of special interest not only in the context of self-defense, but also in relation to the establishment and collapse of the obligate symbiosis with *Symbiodinium*. The coral innate immune repertoire is highly complex and more sophisticated than that of *Hydra* and *Nematostella* (Figure 3) (Shinzato et al., 2011; Hamada et al., 2013). For example, whereas a single canonical Toll/TLR protein is present in *N. vectensis* (Miller et al., 2007), the *Acropora* genome encodes at least four such molecules, as well as five IL-1R-related proteins, and a number of TIR-only proteins (Figure 3A). Likewise, the *Acropora* repertoire of NACHT/NB-ARC domains, which are characteristic of primary intracellular pattern receptors, is again highly complex—an order of magnitude more NACHT/NB-ARC domains are present in coral than in other animals, and some of these cnidarian proteins have novel domain structures.

In the vertebrate innate immune system, ~20 tripartite nucleotide oligomerization domain (NOD)-like receptor proteins that are defined by the presence of NAIP, CIIA, HET-E, and TP1 (NACHT) domains, a C-terminal leucine-rich repeat (LRR) domain, and one of three types of N-terminal effector domain, are known to function as primary intracellular pattern recognition molecules (Figure 3B) (Hamada et al., 2013). Surveying the coral genome demonstrates a larger number of NACHT- and related domain nucleotide-binding adaptors shared by APAF-1, R proteins, and CED-4 (NB-ARC)-encoding loci (~500) than in other metazoans, and also a surprising diversity of domain combinations among coral NACHT/NB-ARC-containing proteins (Figure 3B). N-terminal effector domains include apoptosis-related domains, caspase recruitment domains (CARD), death effector domains (DED), and Death, and C-terminal repeat domains, such as LRRs, tetratricopeptide repeats, ankyrin repeats, and WD40 repeats. Many of the predicted coral proteins that contain a NACHT/NB-ARC domain also contain a glycosyl transferase group 1 domain, a novel domain combination first found in metazoans. Phylogenetic analyses suggest that the NACHT/NB-ARC domain inventories of various metazoan lineages, including corals, are largely products of lineage-specific expansions. Many of the NACHT/NB-ARC loci are organized in pairs or triplets in the *Acropora* genome, suggesting that the large coral NACHT/NB-ARC repertoire has been generated at least in part by tandem duplication (Hamada et al., 2013). In addition, shuffling of N-terminal effector domains may have occurred after diversification of specific NACHT/NB-ARC-repeat domain types. These attributes illustrate the extraordinary complexity of the innate immune repertoire of corals, which may reflect adaptation to a symbiotic lifestyle in a uniquely complex and challenging environment.

### Apoptosis

**Figure 4:**
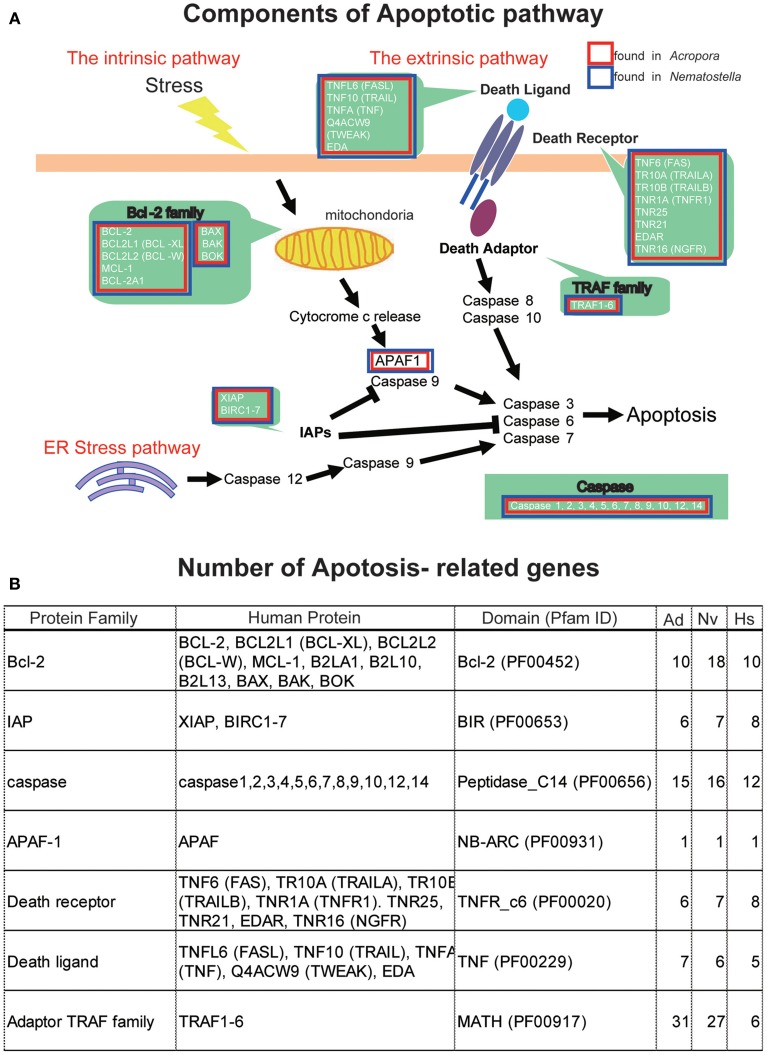
**(A)** Schematic presentation of cellular components involved in the pathways of apoptosis, based on human genes. The extrinsic pathway, intrinsic pathway, and ER stress pathway are three major pathways of apoptosis. Major families are shown by green background. Families found in the *Acropora digitifera* genome are boxed by red and those of *Nematostella vectensis* by blue. **(B)** The number of apoptosis-related family members in the genome of *A. digitifera* (Ad), *N. vectensis* (Nv), and *Homo sapiens* (Hs). The *Acropora* and *Nematostella* genomes contain apoptosis-related genes of which numbers are comparable to those of the human genome, except for a larger number of adaptor TRAF family in the cnidarians.

The apoptotic network of *A. digitifera* is comparable in complexity to those of “higher” animal taxa, including vertebrates (Figure 4A) (Shinzato et al., 2011). Seven Bcl-2 family members containing multiple domains, four IAP family members, 25 caspases, a single APAF-1, four Death receptors, three Death ligands, and 32 members of the TRAF adaptor family are present in the *Acropora* genome (Figure 4B). These numbers are generally comparable to those in the *Nematostella* genome. The TRAF family in *Acropora* and *Nematostella* and the caspases in *Acropora* are overrepresented relative to humans. While no BH3-only members of the Bcl-2 family have been identified (Figure 4B), this may be a consequence of the small size of the BH3 domain and the extent of sequence divergence in these proteins. Failure to detect adaptors with Death domains may reflect the low level of domain conservation characteristic of this family.

### Autophagy

**Figure 5:**
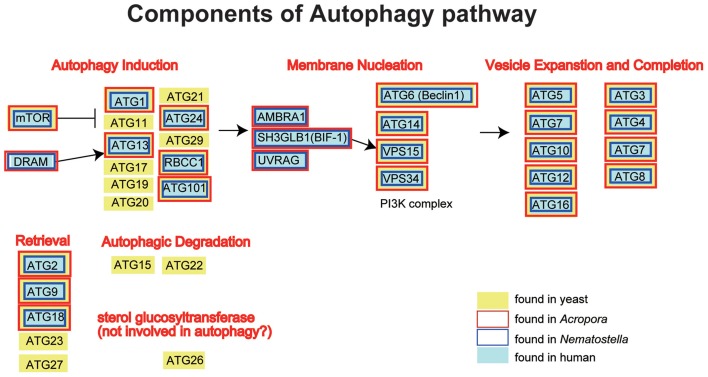
**Schematic presentation of the autophagy pathway, and human (gray backgroud) and yeast genes (*Saccharomyces cerevisiae*; yellow background) involved in the pathway**. The pathway is composed of autophagy induction, membrane nucleation, vesicle expansion and completion, retrieval and autophagic degradation. Genes found in the *Acropora digitifera* genome are boxed by red, those of *Nematostella* by blue. It is obvious that all the human autophagy-related genes have counterparts in *Acropora* and *Nematostella*. In contrast, autophagy-related genes that are found only in the yeast cannot be found in the cnidarian geneomes.

The *A. digitifera* genome contains orthologs of ATG1, ATG2, ATG3, ATG4, ATG5, ATG6, ATG7, ATG8, ATG9, ATG 10, ATG12, ATG13, ATG14, ATG16, ATG18, ATG24, TOR, Vsp34, and Vsp15, but no counterparts of the yeast-specific proteins ATG11, ATG15, ATG17, ATG19, ATG20, ATG21, ATG22, ATG23, ATG26, ATG27, and ATG29 (Shinzato et al., 2011) (Figure 5). The *Acropora* genome also encodes orthologs of human UVRAG, SH3GLB1, DRAM, AMBRA1, RB1CC1, and ATG101 (Figure 5), which are also absent in yeast.

### Genes involved in UV-damage protection

Reef-building corals typically inhabit shallow and relatively clear tropical waters and are therefore constantly exposed to high levels of UV irradiation. Since high solar radiation sometimes causes coral bleaching (Gleason and Wellington, 1993), one intriguing question is how corals protect themselves against UV-damage. UV-absorbing substances potentially act as photoprotective compounds. These include mycosporine-like amino acids (MAAs), scytonemin, carotenoids, and others of unknown chemical structure (Shick et al., 1999; Reef et al., 2009). Although some photoprotective compounds have been isolated from corals (Rastogi et al., 2010), it is often unclear whether symbiotic dinoflagellates and/or bacteria produce the photoprotective compounds, or whether the corals themselves can independently synthesize them.

#### MAAs

**Figure 6:**
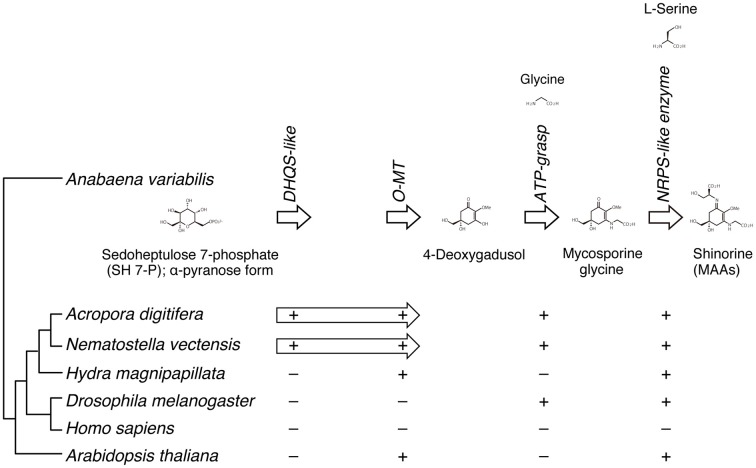
**The genes required for the biosynthesis of shinorine are present in anthozoan cnidarians**. (Upper) The organization of gene cluster involved in the biosynthetic pathway of the photo-protective molecule shinorine, a mycosporine-like amino acid, in the cyanobacterium Anabaena variabilis. (Lower) The presence of corresponding genes in various organisms is indicated by “+.” The Acropora and Nematostella genomes contain homologs of each of the four genes, in which DHQS-like and O-MT are fused each other.

A recent study of the cyanobacterium, *Anabaena variabilis*, identified a four-gene cluster (encoding DHQS-like, O-MT, ATP-grasp, and NRPS-like enzymes) that converts pentose-phosphate metabolites into shinorine, one of MAAs (Figure 6) (Balskus and Walsh, 2010). A search of cnidarian gene models for components of the shinorine gene cluster revealed that this four-gene pathway is present in both *Acropora* and *Nematostella*, but not in *Hydra* (Shinzato et al., 2011). This strongly suggests that both *Acropora* and *Nematostella* can synthesize shinorine by themselves, which may be a precursor for photoprotective compounds.

In addition, molecular phylogenetic analyses show that homologous proteins in *Acropora* have more sequence similarities to those of bacteria and dinoflagellates (Shinzato et al., 2011). These genes might have been acquired via horizontal gene transfer (Starcevic et al., 2008). For example, during the evolution of cnidarian stinging cells, a subunit of bacterial poly-γ-glutamate (PGA) synthase was transferred to an animal ancestor via horizontal gene transfer (Denker et al., 2008). It has been proposed that in marine environments, horizontal gene transfer is important in adapting to ecological vagaries (Keeling, 2009).

#### Scytonemin

**Figure 7:**
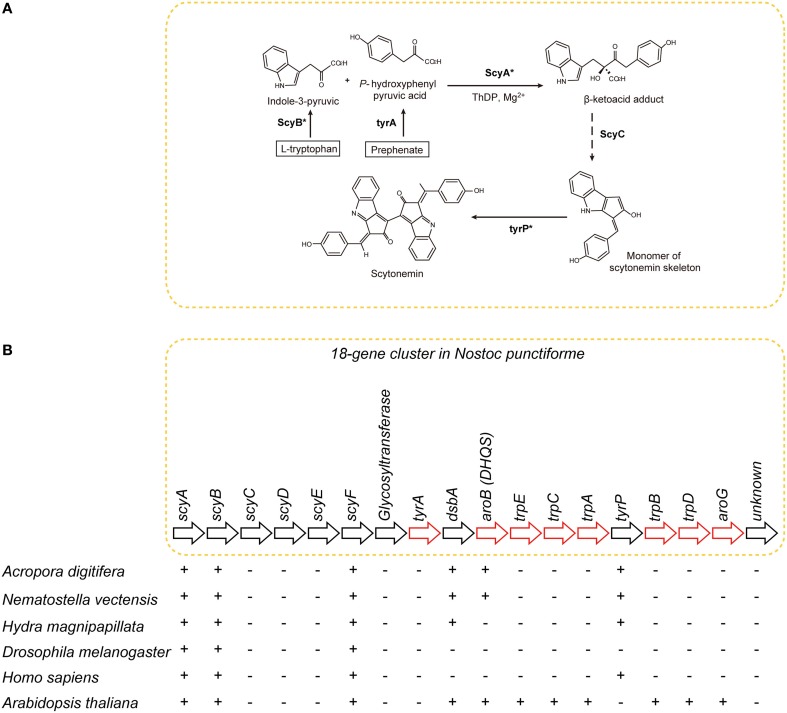
**The distribution of genes associated with biosynthesis of scytonemin in cyanobacteria, cnidarians, and other metazoans. (A)** Pathways of biosynthesis of the photo-protective molecule scytonemin in the cyanobacterium *Nostoc punctiforme* (Soule et al., 2007; Rastogi et al., 2010). Genes encoding the enzymes indicated with asterisks were identified in the *A. digitifera* genome. **(B)** Schematics showing the organization of the scytonemin gene cluster. Genes indicated by red arrows encode enzymes involved in the biosynthesis of aromatic amino acids. The presence of corresponding genes in various organisms is indicated by “+,” indicating that a TBLASTN search against *N. punctiforme* as query showed significant hits. Anthozoan genomes encode a gene, *aroB* homolog, involved in aromatic metabolism that is not found in higher metazoans.

The UV-blocker, scytonemin, is found exclusively in cyanobacteria. In *Nostoc punctiforme*, its biosynthesis is controlled by a cluster of 18 genes (Figure 7) (Soule et al., 2007; Balskus and Walsh, 2008). The cluster comprises one subcluster of genes involved in aromatic amino acid biosynthesis, and a novel subcluster of genes of unknown function (Soule et al., 2009). The former includes *tyrA, dsbA, aroB, trpE, trpC, trpA, tyrP, trpB, trpD*, and *aroG* (Figure 7B). The latter includes *scyA, scyB, scyC, scyD, scyE*, and *scyF* (Figure 7B).

The *A. digitifera* genome contains only six of the 18 genes: namely, *scyA, scyB, scyF, dsbA, aroB*, and *tyrP* (Figure 7) (Shoguchi et al., 2013c). This result suggests that coral cannot synthesize scytonemin independently. Molecular phylogenetic analyses indicate that coral *scyA* and *scyB* are associated with bacterial genes for acetolactate synthase and glutamate dehydrogenase, respectively. This suggests that these enzymes are coupled with PGA/amino acid biosynthesis in corals. In addition, *scyA, scyB*, and *aroB* (*DHQS-like*) are likely to have originated by horizontal transfer from bacteria.

#### Glyoxylate cycle enzymes: malate synthase and isocitrate lyase

Glyoxylate cycle enzymes play a role in lipid metabolism in plant seeds (Kornberg and Beevers, 1957). Although this pathway has not been found in animal lineages, nematode genomes contain genes encoding enzymes involved in the pathway (Liu et al., 1995). Interestingly, the *A. digitifera* genome contains one *isocitrate lyase* (*ICL*) gene and two *malate synthase* (*MS*) genes. Orthology between *Acropora* and *Nematostella* is supported by molecular phylogenetic analysis (Shoguchi et al., 2013c). The genes, *ICL* and *MS1*, are aligned head-to-head in tandem. In addition, by comparisons between neighboring genes, synteny in the region is also conserved. The anthozoan genes form a clade with bacterial *ICL*. Therefore, the origin of anthozoan genes may be different from those of nematode glyoxylate cycle enzymes.

### Fluorescent proteins

Corals exhibit diverse colors, which depend largely on fluorescent proteins (Matz et al., 1999, 2006). Four basic colors of fluorescent proteins present in corals include cyan (CFP), green (GFP), and red (RFP), and a non-fluorescent blue/purple chromoprotein (Kelmanson and Matz, 2003; Field et al., 2006). Fluorescent proteins are usually composed of ~230 amino acids. Corals are able to synthesize several different fluorescent or colored moieties from amino acids within fluorescent proteins, via two or three consecutive autocatalytic reactions. While CFP and GFP possess the same chromophore, individual chromophores can differ dramatically in spectroscopic characteristics (Henderson and Remington, 2005; Lukyanov et al., 2006).

The *A. digitifera* genome contains one, five, one, and three candidate genes for CFP, GFP, RFP, and chromoprotein, respectively, (Shinzato et al., 2012). The CFP and GFP genes are clustered in an ~80-kb genomic region, suggesting that they originated from an ancestral gene by tandem duplication. Since CFP and GFP possess the same chromophore, this gene clustering may provide the first genomic evidence for a common origin of the two proteins. Comparisons of the fluorescent protein genes of closely related coral species suggest an expansion of chromoprotein genes in the *A. digitifera* genome, and of RFP genes in the *A. millepora* genome. RNA-seq analysis shows that *A. digitifera* fluorescent protein genes are expressed during embryonic and larval stages and in adults, suggesting that these genes play a variety of roles in coral physiology.

A wide variety of roles have been attributed to coral fluorescent proteins, including modulating the efficiency of photosynthesis and photoprotection for the symbionts (e.g., Salih et al., 2000) as well as antioxidant functions (Bou-Abdallah et al., 2006; Palmer et al., 2009). Along with cataloging the coral fluorescent protein repertoire, functions of these proteins should be investigated by future studies, especially in the context of molecular mechanisms involved in environmental stress responses of corals, which are associated with collapse of coral-*Symbiodinium* symbiosis.

### Photoreceptors and circadian clock genes

**Figure 8:**
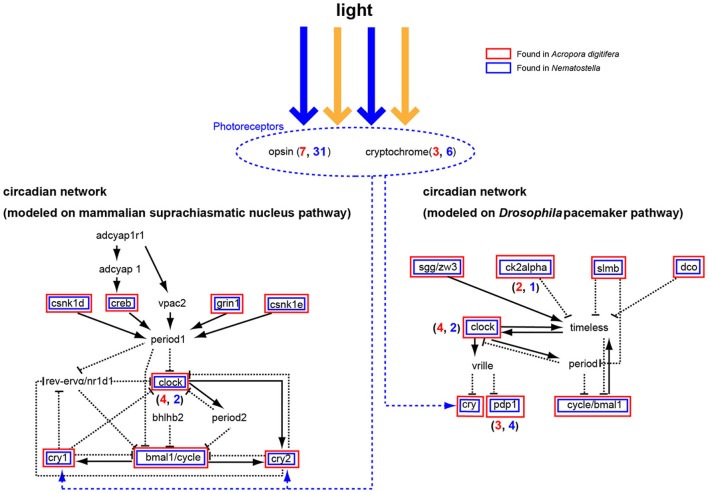
**A circadian network modeled on the mammalian suprachiasmatic nucleus pathway (left) and the *Drosophila melanogaster* pacemaker pathway (right)**. Some cryptochromes detect blue light and function in the network for ~24 h. The *Acropora digitifera* genome contains seven opsin genes and three cryptochrome genes (blue number shows *A. digitifera* gene number; red number, *N. vectensis*). *Acropora* genes in the pathway are shown in red boxes and *Nematostella* genes in blue. The A. digitifera genome is likely to lack period, bhlhb2, vrille, timeless, rev-ervα/nr1d1, adcyap1r1, adcyap1, and vpac2.

Corals exhibit circadian behaviors, which play a pivotal role in timing of spawning. However, little is known about the molecular mechanisms underlying the regulation of these behaviors. Microarray analysis of *Acropora*-*Symbiodinium* suggested complex diel cycles of gene expression (Levy et al., 2011). The *A. digitifera* genome contains seven opsin and three cryptochrome (photoreceptor) genes (Figure 8) (Shoguchi et al., 2013b). Two genes from each family likely underwent tandem duplication in the coral lineage. In addition, *A. digitifera* has orthologs to *Drosophila* and mammalian circadian clock genes: four *clock*, one *bmal/cycle*, three *pdp1-like*, one *creb/atf*, one *sgg/zw3*, two *ck2alpha*, one *dco* (*csnk1d/cnsk1e*), one *slim/BTRC*, and one *grinl* (Figure 8). However, *Acropora* is unlikely to have *vrille, rev-ervα/nr1d1, bhlh2, vpac2, adcyap1, or adcyaplr1* orthologs (Figure 8). Intriguingly, an extensive survey failed to find homologs of *period* and *timeless*, although it found one *timeout* gene. When the coral genes were compared to orthologous genes in *N. vectensis*, a similar repertoire of circadian clock genes was apparent, although *A. digitifera* contains more clock genes and fewer photoreceptor genes than *N. vectensis* (Figure 8). This suggests that the circadian clock system was established in a common ancestor of corals and sea anemones, and diversified by tandem gene duplications and the loss of paralogous genes in each lineage. Future studies should examine how the coral circadian clock functions without *period*.

## Symbiodinium genome

**Figure 9:**
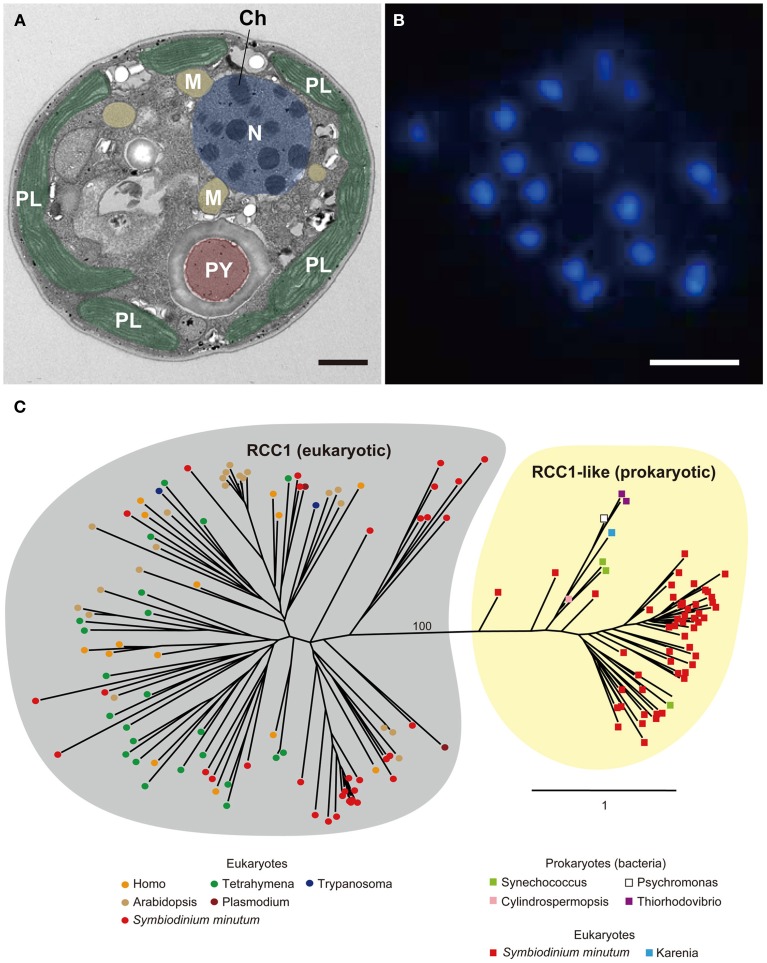
**(A)** Electron micrograph showing permanently condensed chromosomes (Ch) of *Symbiodinium minutum*. The nucleus (N) is shown in purple, plastids (PL) in green, mitochondria (M) in orange, and pyrenoid (PY) in brown. Scale bar, 1 μm. **(B)** DAPI staining of the nucleus showing permanently condensed chromosomes of *S. minutum*. Scale bar, 1 μm. **(C)** RCC1 proteins are eukaryotic proteins that bind to chromatin and play an important role in the regulation of gene expression. A maximum-likelihood phylogeny of 86 RCC1 family proteins encoded in the *S. minutum* genome is shown. The two distinct groupings of eukaryotic RCC1 proteins and prokaryotic RCC1-like proteins are supported by 100% bootstrap duplication. Bar indicates an amino acid substitution per site.

Coral symbionts are all *Symbiodinium* spp. belonging to the phylum Dinoflagellata. Dinoflagellates are unicellular eukaryotes, 10–100 μm in diameter, and characterized by two flagella and a unique cell covering referred to as the theca. Approximately half of them are photosynthetic (Graham and Wilcox, 2000). Dinoflagellates belong to the well-supported Superphylum Alveolata, which also includes ciliates and apicomplexans, such as the malarial parasite, *Plasmodium falciparum* (Burki et al., 2007). Each alveolate lineage has had a distinct evolutionary trajectory with regard to nuclear genome organization, resulting in three divergent outcomes (Gardner et al., 2002; Eisen et al., 2006). Ciliates contain two nuclei, a somatic macronucleus and a micronucleus for reproduction, and they lack plastids. Apicomplexans, due to their parasitic life style in most species, have substantially reduced genomes, with highly degenerate plastids known as apicoplasts (Wilson et al., 1996). Dinoflagellate nuclei have permanently condensed liquid-crystalline chromosomes that lack nucleosomes (Figures 9A,B) (Bouligand and Norris, 2001). In addition, recent studies of partial dinoflagellate genome data show repeated gene copies arranged in tandem arrays (Bachvaroff and Place, 2008), trans-splicing of messenger RNAs (Lidie and van Dolah, 2007; Zhang et al., 2007), and a reduced role for transcriptional regulation, compared to other eukaryotes (Erdner and Anderson, 2006; Moustafa et al., 2010). Given these remarkable characteristics, elucidating the structure and composition of dinoflagellate genomes is essential to understanding their packaging of chromosomal DNA and expression of encoded genes. However, dinoflagellates possess some of the largest eukaryotic nuclear genomes (1500–245,000 megabases [Mbp] in size), which have previously thwarted whole-genome sequencing (Lin, 2011; Wisecaver and Hackett, 2011). In 2013, the genome of a culturable dinoflagellate, *S. minutum*, was decoded (Shoguchi et al., 2013a).

### The nuclear genome

The genome of *S. minutum* is estimated at ~1500 Mbp. Approximately 40-fold coverage of the genome yielded a ~616 Mbp assembly (Shoguchi et al., 2013a). A large quantity of RNA-seq sequences were assembled into 63,104 unique transcripts, 26,691 of which encode complete open reading frames. Gene prediction yielded 41,925 protein models, 77.2% of which (32,366 gene models) are supported by RNA-seq data. In addition, the vast majority of the transcriptome is encoded in the 616-Mbp draft assembly, suggesting that these contigs represent the euchromatin-like region of the *Symbiodinium* genome (http://marinegenomics.oist.jp/genomes/gallery). DNA transposons, retrotransposons, and tandem repeats comprise 0.5, 1.1, and 4.6% of the assembled genome, respectively. The GC-content of the *Symbiodinium* nuclear genome was 44%. This is comparable to GC-content of metazoans and green plants, but contrasts strongly with the AT-rich genomes of other alveolates, such as apicomplexans [*P. falciparum*, 19% GC (Gardner et al., 2002)] and ciliates [*Tetrahymena thermophile*, 22% GC (Eisen et al., 2006)], respectively.

#### Gene content of the dinoflagellate genome

Of 41,925 gene models, 20,983 (50%) encode proteins with known domains. One of the largest dinoflagellate protein families is the EF-hand family, a large family of calcium-binding proteins characterized by a helix-loop-helix structural domain. The second largest dinoflagellate family contains ankyrin repeats, one of the most common protein-protein interaction motifs in nature. When the *Symbiodinium* gene families are compared with those of other eukaryotes, *Symbiodinium* shares a considerable number of homologous genes with *Homo* and *Arabidopsis*, although ~46% of predicted proteins are novel or S*ymbiodinium*-specific.

#### Specific gene expansion in the symbiodinium genome

Dinoflagellates have been predicted to possess 38,000–87,000 protein-coding genes (Hou and Lin, 2009). The presence of a larger number of genes in the *S. minutum* genome (41,925) is likely caused by lineage-specific expansion of genes by duplication (Hou and Lin, 2009). Orthologous gene clustering analyses indicate that 1064 groups (10,912 genes) in the *Symbiodinium* genome have likely resulted from such events. One striking finding is that the regulator of chromosome condensation family protein (RCC1) is highly expanded (discussed below). Calcium channel and calmodulin families are also expanded. Because the largest domain was the EF-hand subgroup of calcium-binding proteins, Ca^2+^ metabolism is clearly of great importance in *Symbiodinium*.

#### Molecular basis of permanently condensed chromatin

As mentioned above, dinoflagellate nuclei are characterized by permanently condensed, liquid-crystalline chromosomes (Figures 9A,B), and dinoflagellate chromosomal organization is a fundamental issues that is still not fully understood (Lin, 2011). In eukaryotes, histone proteins are involved in chromatin modulation, whereas in prokaryotes, histone-like proteins serve this function. The *S. minutum* genome contains both eukaryotic histone genes and prokaryotic histone-like genes, although orthologs of histone H1 are not found in the genome (Shoguchi et al., 2013a). All four core-histone genes (H2A, H2B, H3, and H4) are duplicated. In addition, there are 15 histone-like proteins similar to those found in bacteria.

In addition to enlargement of the genome, a dinoflagellate, *Hermatodinium* sp., gains a novel family of nucleoproteins from an algal virus, termed dinoflagellate/viral nucleoprotein (DVNP) (Gornik et al., 2012). The *Symbiodinium* genome contains 19 genes that appear homologous to DVNPs, suggesting a role for this type of protein in *Symbiodinium* chromosome structure.

The RCC1 proteins (RCC1 superfamily in eukaryotes and RCC1-like repeat proteins in both prokaryotes and eukaryotes) bind to chromatin and play an important role in the regulation of gene expression (Dasso, 1993). As mentioned above, genes for RCC1 have the third highest degree of expansion in the *Symbiodinium* genome, and a total of 189 genes are present in the *Symbiodinium* genome (Shoguchi et al., 2013a). When 86 of these proteins are used for molecular phylogenic analyses, two distinct clusters become evident. One, with 34 *Symbiodinium* proteins consists of those orthologous to eukaryotes, including alveolates, plants, and animals (Figure 9C, left), whereas the other includes 52 proteins with similarities to prokaryotes, including cyanobacteria and proteobacteria (Figure 9C, right). This result potentially explains the characteristic architecture of dinoflagellate chromosomes, although the manner in which they interact with each other to establish and maintain the permanently condensed chromosomes remains to be studied.

#### Unique spliceosomal splicing

**Figure 10:**
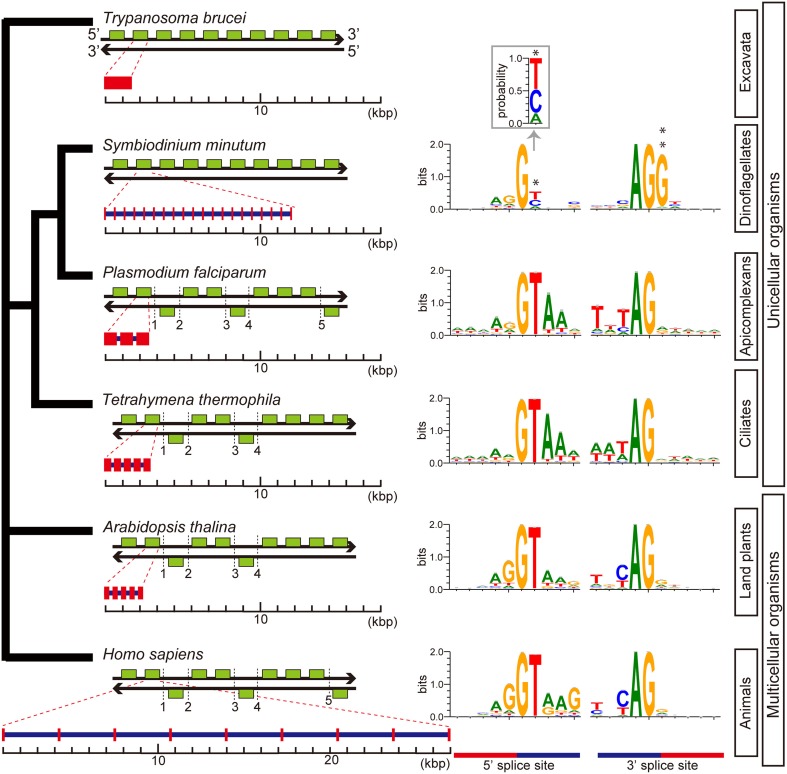
**Architecture of genes and splice site motifs in the nuclear genomes of representative eukaryotes and alveolates**. Green boxes indicate typical patterns of 10-gene arrangements with the number of strand switch regions (SSRs), although the SSRs shown here are not always typical. Patterns are based on the analyses shown in Figure 6. Gene architecture shows average gene lengths (exons in red and introns in blue) with the average intron number per gene. The sequence motif of the splice site is illustrated using WebLogo. Only two genes with spliceosomal introns in *Trypanosoma brucei* have been reported, but the motif was not shown. The unusual gene organization on the same strand of DNA shows similarities between *Symbiodinium* and *Trypanosoma*. Additionally, analyses of intron-richness and the weakness of 5′ splice site signals (asterisk) indicate that *Symbiodinium* has the most unusual genome organization found in a eukaryote genome to date. The probability of position 2 at the 5′ splice site is shown in inset. A double asterisk shows G conserved at the 3′ splice site.

Although previous reports have suggested that introns are relatively uncommon in dinoflagellate genes (Okamoto et al., 2001; Hoppenrath and Leander, 2010), genes of *S. minutum* are highly intron-rich. 39,970 of the 41,925 genes (95%) are composed of multiple exons. The average number of exons per gene reaches 19.6, and some genes contain more than 200 introns (Shoguchi et al., 2013a). In addition, spliceosomal introns of *Symbiodinium* are unique among eukaryotic genomes. In other eukaryotes, introns are excised under the GT-AG rule, wherein GT and AG are used as recognition nucleotides at 5' and 3' splice sites, respectively, (Figure 10). In contrast, *Symbiodinium* uses GC and GA at the 5' donor splice site, in addition to GT (Figure 10). GC usage frequency is nearly equal to that of GT. The presence of these 5' splice sites provides the first evidence in eukaryotes that the majority of mRNA splicing does not always follow the GT-AG rule. Another feature of *Symbiodinium* splicing is that the 3' acceptor splice site, AG, is frequently followed by the nucleotide G (Figure 10), although a similar phenomenon is known in human minor alternative splice sites (Thanaraj and Clark, 2001).

Key steps in RNA splicing are performed by spliceosomes, acting in concert with five small nuclear RNA molecules (snRNAs; *U1, U2, U4, U5*, and *U6*). The five major snRNAs recognize nucleotide sequences that specify where splicing is to occur, and they participate in spliceosome chemistry (Rogozin et al., 2012). In the *Plasmodium* and *Tetrahymena* genomes, snRNAs are scattered throughout the genome, whereas in metazoans and green plants, two different types of the five major snRNAs are sometimes tandemly aligned (Wang and Brendel, 2004; Marz et al., 2008). In contrast, in the *Symbiodinium* genome, all five snRNAs, *U1, U2, U4, U5*, and *U6* occur in a cluster, in addition to other snRNAs scattered across about 70 locations. This is the first discovery of an snRNA gene cluster in a eukaryote genome. It has been reported that *trans*-splicing of messenger RNAs is common in dinoflagellates (Lin, 2011; Wisecaver and Hackett, 2011). The *Symbiodinium* genome contains spliced-leader (SL) genes with a conserved SL sequence.

#### Unique arrangement of genes in the genome

**Figure 11:**
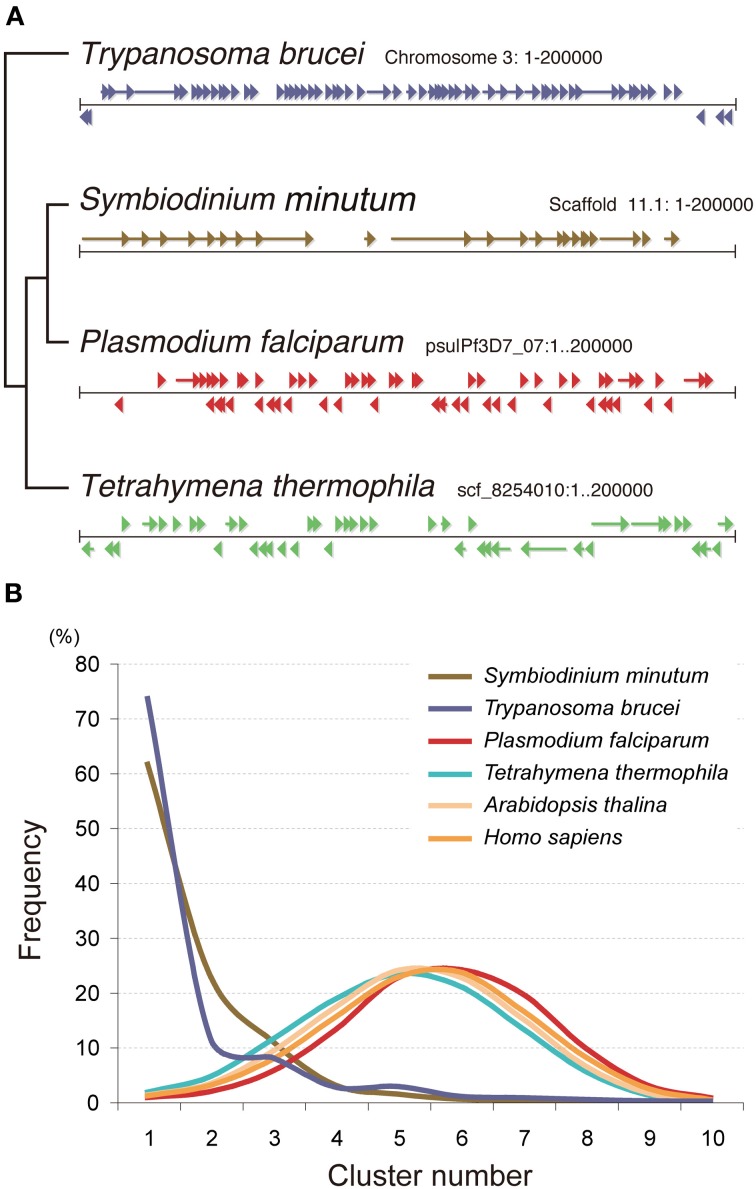
**Nuclear gene arrangement in the *Symbiodinium minutum* genome. (A)** Examples of gene arrangement in the 200-kbp nuclear genome are shown and compared among *Symbiodinium minutum* (dinoflagellate), *Trypanosoma brucei* (euglenozoan), *Plasmodium falciparum* (apicomplexa), and *Tetrahymena thermophila* (ciliate). In contrast to the genomes of *Tetrahymena, Plasmodium*, and *Acropora*, that show a random arrangement of protein-coding genes (arrowheads and arrows), the genomes of *S. minutum* and *Trypanosoma* are arranged into a large directional gene cluster in a head-to-tail orientation. **(B)** A search of the directional gene cluster using a 10-gene window shows the strong tendency toward unidirectional alignment of genes in the *S. minutum* and *Trypanosoma* genomes. Each line represents a frequency histogram for changes in the gene orientation between successive genes in the genome. The X-axis represents the number of orientation changes as one moves through windows of 10 genes. For examples as indicating random orientation, the poisson distributions with μ = 4.5 (average) and 0.2 are shown.

The *Symbiodinium* genome is also unique in the context of gene arrangement (Shoguchi et al., 2013a). In contrast to the random arrangement of protein-coding genes in the genomes of *Tetrahymena, Plasmodium, Arabidopsis*, and *Homo*, those of the *Symbiodinium* and *Trypanosoma* genomes show a clear tendency for tandem and unidirectional gene alignment. The grade of change in gene direction was searched using a 10-gene sliding window (Figure 11). Graphs of these data for *Plasmodium, Tetrahymena, Arabidopsis*, and *Homo* show a peak between 4 and 5 changes in orientation, indicating the frequency of strand switch regions (SSRs) between genes in head-to-head or tail-to-tail orientations (Figure 11). In contrast, *Symbiodinium* and *Trypanosoma* show a cluster (Figure 11). This indicates a strong tendency for tandem alignment of genes or clustering of unidirectionally aligned genes in the *Symbiodinium* and *Trypanosoma* genomes.

#### Genes involved in the basic transcriptional machinery

Although the *S. minutum* genome is unique in regard to permanently condensed chromosomes, spliceosomal splicing, and unidirectionally aligned genes, the genome contains highly conserved basic transcriptional machinery components, including RNA polymerase I, II, and III, basal transcription factors, such as TFIID and TATA-binding protein (TBP), and transcription elongation factors (Shoguchi et al., 2013a). In contrast, the genome contains a few sequence-specific transcription factors, including 19 gene models with AP2 domain(s), 15 models with HMG-box domain(s), eight models with zf-C2H2 domain(s), and others. These results suggest constant, steady transcription of *Symbiodinium* genes with fewer genes under sequence-specific transcriptional control.

### Chloroplast (plastid) genome

Chloroplasts (plastids) are common photosynthetic organelles in eukaryotic algae and land plants. Plastids first may have arisen when non-photosynthetic eukaryotic hosts acquired cyanobacterial endosymbionts by a process termed “primary endosymbiosis” (Howe et al., 2008; Keeling, 2010). Other non-photosynthetic eukaryotes may have subsequently acquired endosymbionts from photosynthetic eukaryotes to create secondary plastids (Howe et al., 2008; Keeling, 2010). In some lineages including dinoflagellates, secondary plastids may have been lost and replaced with secondary endosymbiotic plastids or other primary endosymbiotic plastids, resulting in tertiary plastids (Allen et al., 2011).

Evolutionary changes in plastid genomes in alveolates are dramatic. Ciliates lost plastids and became heterotrophic, while parasitic apicomplexans retain unpigmented plastid remnants termed apicoplasts. On the other hand, two species closely related to apicomplexans, *Chromera velia* and *Vitrella brassicaformis*, are photosynthetic. Their plastid genomes retain ancestral characteristics of both apicomplexan and dinoflagellate plastids and probably share a common red algal endosymbiont (Janouskovec et al., 2010). Interestingly, rapidly evolving dinoflagellate plastids show a great variety of reduced stages. Their gene content has been dramatically diminished by large-scale transfer of genes to the nucleus, leaving only 12–17 genes in the plastids (Howe et al., 2008). Conventional plastid genomes have all genes physically linked in one molecule, typically 120–200 kb in size (Keeling, 2010), while dinoflagellate plastid genes reside on small plasmids of 2.2–6 kb, termed “minicircles” (Zhang et al., 1999), containing a few genes and a core, non-coding region, which is conserved within species and plays a regulatory role (Zhang et al., 2002; Leung and Wong, 2009; Wisecaver and Hackett, 2011). Moreover, a number of unusual post-transcriptional RNA modifications, including the addition of 3′ terminal poly(U)tracts, occur in the ancestral chloroplasts of dinoflagellates. Extensive RNA editing occurs in some dinoflagellates (Zauner et al., 2004; Wang and Morse, 2006; Dang and Green, 2009), employing diverse editing types that have not been observed in mammals and plants. This leads to speculation about the functional connection between poly(U)tailing and RNA editing in dinoflagellate plastid transcripts (Dang and Green, 2009).

In *S. minutum*, 95 of 109 plastid-associated genes have been transferred to the nuclear genome and subsequently expanded by gene duplication (Mungpakdee et al., 2014). Only 14 genes remain in plastids, as DNA minicircles. Each *Symbiodinium* minicircle (1.8–3.3 kb) contains one gene and a conserved non-coding region containing putative promoters and RNA-binding sites. Nine types of RNA editing, including a novel G/U type, are evident in minicircle transcripts, but not in genes transferred to the nucleus. In contrast to DNA editing sites in dinoflagellate mitochondria, which tend to be highly conserved across all taxa, editing sites employed in DNA minicircles are highly variable from species to species. Editing is crucial for core photosystem protein function. It restores evolutionarily conserved amino acids and increases peptidyl hydropathy. RNA editing is also likely to increase protein plasticity necessary to initiate photosystem complex assembly.

### Mitochondrial genome

In most metazoans, mitochondrial genomes are 13–20-kb, compact, circular molecules, containing 12–13 proteins, 24–25 tRNAs, and 2 rRNAs. As in the case of plastid genomes, mitochondrial genomes also dramatically changed during evolution. Ciliates (*Tetrahymena* and *Ichthyophthirius*) have linearly mapped mitochondrial genomes of 43 kb with a normal gene number (Burger et al., 2000), while only 3 protein-coding genes and fragmented rRNAs organized as part of linear repeats of about 6–7 kbp are found in parasitic apicomplexans (*Plasmodium, Babesia*, and *Theileria*) (Hikosaka et al., 2012). Gene content of dinoflagellate mitochondrial genomes is comparable to that of apicomplexans (Slamovits et al., 2007), but with highly fragmented and rearranged genome structure (Waller and Jackson, 2009).

A 49-kmer assembly of only high coverage (>100) Illumina paired-end reads of a dinoflagellate, *S. minutum*, revealed two candidate mitochondrial scaffolds, two linear DNAs (19,577 and 291,368 bp) (Mungpakdee et al., unpublished data). Blast and transcriptome mapping show that one contains only *cox1* and the other *cob, cox3*, and 6 fragmented of large subunit (LSU) rRNA genes. Fragments of small subunit (SSU) rRNA and tRNA genes are not found in the *Symbiodinium* mitochondrial genome. The evolution of the mitochondrial genome in *Symbiodinium*, as well as in other dinoflagellates requires further investigation to reach some consensus.

## Conclusion

Genomic information is essential for future studies of molecular and cellular mechanisms underlying the establishment, maintenance, and breakdown of obligate endosymbiosis of corals with photosynthetic dinoflagellates *Symbiodinium*. In general, the coral genome is unique in that frequent horizontal gene transfer is evident in UV-protection genes. In addition, *Symbiodinium* is one of diverse dinoflagellates in regard to nuclear, plastid, and mitochondrial genomes. At present, many questions about endosymbiosis remain to be answered, but genomic information will greatly facilitate future studies of coral-dinoflagellate endosymbiosis.

### Conflict of interest statement

The authors declare that the research was conducted in the absence of any commercial or financial relationships that could be construed as a potential conflict of interest.
